# A unique *in vivo* experimental approach reveals metabolic adaptation of the probiotic *Propionibacterium freudenreichii* to the colon environment

**DOI:** 10.1186/1471-2164-14-911

**Published:** 2013-12-23

**Authors:** Taous Saraoui, Sandrine Parayre, Grégory Guernec, Valentin Loux, Jérôme Montfort, Aurélie Le Cam, Gaëlle Boudry, Gwenaël Jan, Hélène Falentin

**Affiliations:** 1 INRA, UMR1253, Science et Technologie du Lait et de l’Œuf, F 35042 Rennes, France; 2 AGROCAMPUS OUEST, UMR1253, Science et Technologie du Lait et de l’Œuf, F 35042 Rennes, France; 3 INRA, UR1037, Laboratoire de Physiologie et Génomique des Poissons, F 35000 Rennes, France; 4 INRA, UR1077, Mathématique, Informatique et Génome, F 78352 Jouy-en-Josas, France; 5 INRA, UR1341, Alimentation Adaptions Digestives, Nerveuses et Comportementales, F 35590 St-Gilles, France; 6 IFREMER, Laboratoire de Science et Technologie de la Biomasse Marine, Nantes, France; 7 INSERM, UMR 1027 Toulouse, France

## Abstract

**Background:**

*Propionibacterium freudenreichii* is a food grade bacterium consumed both in cheeses and in probiotic preparations. Its promising probiotic potential, relying largely on the active release of beneficial metabolites within the gut as well as the expression of key surface proteins involved in immunomodulation, deserves to be explored more deeply. Adaptation to the colon environment is requisite for the active release of propionibacterial beneficial metabolites and constitutes a bottleneck for metabolic activity *in vivo*. Mechanisms allowing *P. freudenreichii* to adapt to digestive stresses have been only studied *in vitro* so far. Our aim was therefore to study *P. freudenreichii* metabolic adaptation to intra-colonic conditions *in situ*.

**Results:**

We maintained a pure culture of the type strain *P. freudenreichii* CIRM BIA 1, contained in a dialysis bag, within the colon of vigilant piglets during 24 hours. A transcriptomic analysis compared gene expression to identify the metabolic pathways induced by this environment, versus control cultures maintained in spent culture medium.

We observed drastic changes in the catabolism of sugars and amino-acids. Glycolysis, the Wood-Werkman cycle and the oxidative phosphorylation pathways were down-regulated but induction of specific carbohydrate catabolisms and alternative pathways were induced to produce NADH, NADPH, ATP and precursors (utilizing of propanediol, gluconate, lactate, purine and pyrimidine and amino-acids). Genes involved in stress response were down-regulated and genes specifically expressed during cell division were induced, suggesting that *P. freudenreichii* adapted its metabolism to the conditions encountered in the colon.

**Conclusions:**

This study constitutes the first molecular demonstration of *P. freudenreichii* activity and physiological adaptation *in vivo* within the colon. Our data are likely specific to our pig microbiota composition but opens an avenue towards understanding probiotic action within the gut in further studies comparing bacterial adaptation to different microbiota.

## Background

*Propionibacterium freudenreichii* is a food-grade GRAS bacterium, used as a cheese ripening starter, which displays promising probiotic potential that needs to be explored more deeply [1]. *In vivo* experiments indicate that *P. freudenreichii* consumption results in modulation of the gut microbiota, including enhancement of the bifidobacterial population [2-4] and decrease in *Clostridium* and *Bacteroides*[5]. Modulation of gut content enzymatic activity was also reported, including enhancement of beta-galactosidase activity and decrease in beta-glucuronidase and azoreductase activities [6-8]. *In vitro* and in human-microbiota-associated (HMA) rats, *P. freudenreichii* favored apoptotic depletion of colon cancer cells [9,10]. Finally, *P. freudenreichii* was shown to induce the synthesis of the regulatory cytokine IL-10 in human PBMCs and to protect mice against experimental colitis [11]. In humans, a milk whey culture of *P. freudenreichii* correspondingly alleviates ulcerative colitis symptoms [12,13].

There is experimental evidence that the aforementioned probiotic effects rely on the active release of beneficial metabolites within the gut. The bifidogenic effect of *P. freudenreichii* depends on production of propionate, 2-amino-3-carboxy-1,4-napthoquinone (ACNQ) and 1,4-dihydroxy-2-naphthoic acid (DHNA), an intermediate in the menaquinone (vitamin K2) biosynthesis pathway [14]. ACNQ, which may derive from DHNA, serves as an electron acceptor of NAD(P)H diaphorase and as electron donor of NAD(P)H peroxidase in bifidobacteria [15,16]. NAD(P) + regeneration would be responsible for bifidobacteria growth stimulation by propionibacteria via DHNA and ACNQ. Production of bacteriocins by dairy propionibacteria may also participate in microbiota modulation [17]. Dairy propionibacteria also release beta-galactosidase in the presence of bile salts [12]. The modulation of important parameters of gut physiology such as motility, absorption, differentiation and apoptosis depends on propionate, folate (vitamin B9) and nitric oxide (NO) production [1,18]. Finally, protective properties of *P. freudenreichii* against colitis are reportedly linked with the ability to synthesize immunomodulatory proteins [11] and to release the bifidogenic compounds cited above [19,20].

Adaptation to the colonic lumen conditions is a prerequisite to active production and release of beneficial metabolites. However, this environment may be stressful for ingested bacteria, due to host defense mechanisms including pH variations, peristaltism, antimicrobial peptides and bile acids, and to competition with resident microbiota for nutrient acquisition and for growth niches. Adaptation to the two major digestive stresses, acidic pH and elevated bile salts concentration, was demonstrated *in vitro* in *P. freudenreichii* and the corresponding mechanisms were investigated using proteomics. Acid adaptation requires induction of enzymes involved in DNA synthesis and repair, in central carbon metabolism, including the transcarboxylase cycle specific to propionic fermentation in propionibacteria; and in polypeptide metabolism (ClpB, ClpC) as well as the universal chaperones GroEL and GroES [21-23]. Bile salts adaptation relies on the induction of proteins involved in stress sensing and signal transduction, in oxidative stress remediation and detoxification (superoxide dismutase, cysteine synthase, ABC transporters) and in the Wood-Werkman cycle [23,24]. Such adaptation leads to *P. freudenreichii* tolerance to elevated doses of these stresses. Moreover, *P. freudenreichii* can survive and maintain an active metabolism within the digestive tract of HMA rats [9,25] and of human volunteers [10,26]. However the molecular mechanisms responsible for this adaptation have not been fully elucidated in *P. freudenreichii*.

To investigate probiotic activity within the colon environment, i*n vivo* expression technology (IVET) and recombination-based *in vivo* expression technologies (R-IVET) can be used. R-IVET achieved to compare gene expression between laboratory medium and *in vivo* conditions [27]. However, such methods require a promoter probe library in a genetically accessible probiotic strain, which is not available for *P. freudenreichii*. Moreover, they allow detection of promoter activities and require confirmation of gene induction. Another approach is thus needed for genome-wide expression monitoring in this actinobacterium. We recently developed proteomic and transcriptomic tools to monitor *in vitro* gene expression under different stress conditions including heat, cold, acid, bile salts or conditions encountered within cheese curd [21,23,24,28-30]. For the present work, we developed a new experimental strategy to investigate *P. freudenreichii* activity within the colon of live animals. We chose the pig, a suitable model, because of the physiological and anatomical similarities of its gastro-intestinal tract to that of humans [31]. A stationary phase culture, contained in an implant that allowed exchange with the luminal content through a dialysis membrane, was kept within the colon of vigilant piglets during 24 hours. Using our transcriptomic tools, bacterial gene expression was then compared to that of control bacteria kept in spent YEL culture medium.

## Methods

### Strain culture

This study was conducted on *P. freudenreichii* strain CIRM BIA 1 whose genome has been sequenced and annotated [32]. It was obtained from the CIRM BIA collection (Centre International de Ressources Microbiennes – Bactéries d'Intérêt Alimentaire, STLO, INRA Rennes, France) and was grown at 30°C in YEL broth [33] in closed glass tubes without agitation. Growth was monitored spectrophotometrically at 650 nm (A_650nm_). Cultures were stopped at an A_650nm_of 3 ; *i.e.* in early stationary phase (close to 72 hours). Cultures were either kept in the spent YEL culture medium for 24 hours or concentrated 5 times by a 5 min centrifugation (6000 g) at the growing temperature (30°C) and resuspended in spent YEL culture medium prior to introduction into dialysis tubing.

### Intra-colonic dialysis tubing

An intra-colonic dialysis tubing designed to contain the bacteria but allow the exchange of solutes was custom-made using a 50 kDa-cut-off SpectraPore® regenerated cellulose dialysis tube, 1 cm in diameter. This tube (13 cm long) was sealed at one end by a knot and fastened to a surgical catheter at the other end. Tightness was achieved using surgical thread (Additional file 1: Figure S1). Sterilization of the dialysis tubing was performed for 10 min at 105°C. Water tightness was checked before use. Bromophenol Blue permeability and Dextran Blue impermeability were checked, along with the diffusion of bile salts across the dialysis tubing envelope (see Additional file 1: Figure S1). Five mL of stationary phase culture (10^10^ CFU/mL) were introduced aseptically in the dialysis tubing through the catheter using a syringe equipped with a 0.8 mm-diameter needle.

### Animal procedure

The experimental protocol was designed in compliance with recommendations of the French law (2001–464 29/05/01) and EEC (86/609/CEE) for the care and use of laboratory animals. It was approved by the Comité Rennais d’Ethique en matière d’Expérimentation Animale (protocol #R-2010-GB01). Four ((Pietrain x Landrace) x Large White) pigs (30 kg) from the experimental herd of INRA St-Gilles (France) were housed individually in stainless steel cages in a temperature-controlled (23°C) and 12 h/12 h dark/light cycle room. They were acclimated to their new housing conditions for 1 week. After an overnight fast, pigs were anesthetized using isoflurane. After laparotomy, a silicon T-cannula was inserted into the proximal colon (15 cm distal to the ileo-caecal valvula) and exteriorized on the left side of the animal. Pigs were given morphine chlorhydrate subcutaneously during the surgical procedure and 12 hr after as well as ampicillin (30 mg/kg/d intra-muscular) for 3 days. Animals allowed one week for recovery and were fed a standard pig diet (Additional file 2: Table S1). After this recovery phase, the dialysis tubing was inserted into the colonic lumen through the cannula and left for 24 hrs. Briefly, animals were sedated using ketamine (10 mg/ intra-muscular). The cannula was opened and the dialysis tubing inserted slowly into the colonic lumen. It was tightly anchored to the cannula using a thread. The cannula was then closed. The pig was monitored while recovering from sedation then allowed freedom of movement and normal access to food and water for 24 hrs. After this period, the animal was again sedated with ketamine and the dialysis tubing slowly removed from the colonic lumen by pulling on the thread. A new dialysis tubing was then inserted. This was repeated 4 times on 4 consecutive days, to generate 16 biological replicates from 16 independent fresh cultures. A flowchart (Additional file 3: Figure S2) summarized the complete experiment.

### RNA extraction and quality control

1 ml of each culture or of a 1/10 v:v PBS dilution of the content of the dialysis tubing was mixed to 2 volumes of RnaProtect (Qiagen, Hilden, Germany), left 5 min at room temperature and then centrifuged (8,000 g, 10 min, at room temperature). The supernatant was removed and the pellet stored at −80°C until total RNA was extracted. Pellets were thawed on ice, suspended in 200 μL of lysis buffer (50 mM Tris–HCl, 1 mM EDTA; pH 8.0) containing 20 mg/mL lysozyme (MP Biomedicals, Illkirch, France) and 50 U/mL mutanolysin (Sigma, Saint Quentin Fallavier, France), and incubated for 15 min at 24°C. The suspensions were then transferred to two milliliters tubes containing 50 mg of zirconium beads (diameter, 0.1 mm; BioSpec Products, Bartlesville, OK) and 100 μL of SDS (10%). The tubes were shaken twice for 90 s at 30 Hz with a bead beater (MM301; Retsch, Haan, Germany) chilled on ice for two min between the shaking steps. RNA extraction was then performed using an RNeasy minikit (Qiagen) and the Qiacube extraction robot (Qiagen) according to the instructions of the manufacturer. RNA were suspended in 50 μL of RNase-free water and treated with DNase (DNA free; Ambion, Cambridgeshire, United Kingdom) according to the instructions of the supplier, and then stored at −80°C until use. Quantification of RNA was performed and contamination of RNA by proteins was assessed spectrophotometrically using a NanoDrop ND-1000 spectrophotometer (NanoDrop Technologies,Inc., Rockland, DE). RNA quality was evaluated using an Agilent 2100 bioanalyzer (Agilent Technologies, Santa Clara, CA, USA). All of the RNA samples from spent medium had a RIN value greater than 7.5, indicating good integrity of the rRNA. On the other hand, three samples from the intra-colonic environment was excluded from hybridization experiments because its RIN values was lower than 7.5 and one sample from the intra-colonic environment was excluded because its RNA concentration was too low (for details on RIN and concentration see Additional file 4: Table S2). The absence of genomic DNA was confirmed by quantitative PCR.

### Microarray hybridization

A 8 × 15 K microarray was designed using eArray (Agilent technologies (https://earray.chem.agilent.com/earray/)) with the following parameters: probe length 60 bp, 1 probe per target, probe orientation sense, best probe methodology, design with 3’ bias, possibility of probes trimming, preferred Tm 85°C (isothermal and GC rich genome). Microarrays are available under design ID 034169 and array name pfreudenreichii-cirm1. RNA were retrotranscribed into cDNA and labelled with cyanine with the One color microarray-based prokaryote analysis FairPlay III Labeling kit (Agilent Technologies) according to manufacturer instructions. Briefly, for each sample, 6 μg of total RNA were retrotranscribed into cDNA with incorporation of an amino-allyl dUTP and labeled using CyDye Cy3 mono reactive (GE Healthcare, Orsay, France). Yield (> 650 ng of cDNA) and specific activity (> 40 pmol of Cy3 per μg of cDNA) of the Cy3-cDNA produced were checked with the Nanodrop. 600 ng of Cy3-cDNA were hybridized on a sub-array. Hybridization was carried out for 17 hours at 65°C using a Microarray hybridization chamber (Agilent Technologies) in a rotating hybridization oven prior to washing per Agilent protocol and scanning with an Agilent Scanner (DNA Microarray Scanner, Agilent Technologies) using the standard parameters for a gene expression 8 × 15K oligoarray (5 μm and 20 bits). Data were then obtained with the Agilent Feature Extraction software (10.5.1.1) according to the appropriate GE protocol (GE1_105_Dec08). Probes were considered valid when the corresponding spots were present in at least 80% of the replicates of each experimental condition after the flagging procedure (66 probes were thus omitted from the rest of the analysis). The remaining bad spots (less than 5% of the total) were imputed by the k-nearest neighborhood (k = 4) approach [34].

### Retrotranscription quantitative PCR

In order to confirm the results from the transcriptomic analyses, reverse transcription quantitative PCR (RT-qPCR) experiments were carried out. The use of 3 biological RNA replicates for each condition has been used in this work as a control to confirm transcriptomic results obtained using microarrays on 8 biological replicates. The RT-qPCR were performed on RNA which were in sufficient amount to perform PCR targeting 42 genes observed as differentially expressed in the microarray analysis. Primers were designed using Primer3 Plus software [35] with default parameters except for the difference of melting temperature between the forward and reverse primers set to less than 1°C. The primer sequences for the tested genes are detailed in Additional file 5: Table S3. cDNA synthesis, quantitative PCR, and cycle thresholds (Ct) were performed according to Falentin et al. [36]. Briefly, cDNA was synthesized using a qScript TM cDNA synthesis kit (Quanta BioSciences, Maryland, USA). Amplification by qPCR was performed with a 15 μL final volume mixture containing 5 μL of a cDNA template diluted 1 to 40, 0.5 mM of each primer and 16 IQTM SYBR GreenSupermix (BioRad, California, USA), in an Opticon 2 real-time PCR detector (Biorad). Three cDNA obtained from spent YEL medium culture conditions (corresponding to three biological replicates) and three cDNA obtained from the intra-colonic environment (corresponding to three biological replicates) were amplified. Amplification cycles consisted of an initial step at 95°C for 5 min followed by 40 cycles of 95°C for 30 s, 60°C for 30 s, and 72°C for 1 min. An amplicon denaturation step of 0.5°C/min from 65°C to 90°C was performed to verify amplification specificity and determine amplicon melting temperature.

Genes that were not differentially expressed in the microarray analysis were chosen as possible internal standards for RT-qPCR normalization. The stability of mRNA expression of three genes: *serA*, *cbiM, ndk* was checked using the geNorm VBA applet for Microsoft Excel [37] and were chosen as internal standard for normalization.

### Statistical analysis of transcriptomic data

The aim of the statistical analysis was to generate the most reliable list of genes with significantly different levels of expression between our two distinct conditions: the intra-colonic environment and the YEL medium.

An analysis of the variability within and between piglets (N = 13770 probes) using matrix of parametric correlations allowed us to choose the two most reproducible replicates of 4 per piglet (Additional file 6: Figure S5). Four independent signals (one per piglet) were then obtained by averaging the two selected signals from each piglet. For the YEL condition, 8 of 12 biological replicates were randomly selected and averaged two by two, establishing 4 independent measurements (for details see Additional file 4: Table S2).

The signals corresponding to the same probes were averaged, and the log2-signals were subsequently scale normalized using the median value of each array [38] given a final data sets of 2229 genes.

Differential comparisons between groups were performed gene by gene using modified *t*-test with limma adjustment (p-value ≤ 10^-4^) from the limma package of R software [39,40]. The Bayesian approach of which remains very convenient and a powerful tool when working with a low number of biological replicates [41].

The increase in the type I error rate induced by the multiplicity of tests was controlled by the Benjamini-Hochberg adjustment from the False Discovery Rate family [35]. Genes were declared as differentially expressed with P ≤ 0.001 and |fold change| > 2.

For the RT-qPCR analysis, geNorm was used to determine the normalized expression level of genes of interest (http://medgen.ugent.be/genorm/). A student *T*-test was performed and changes in gene expression between the two conditions with a P-value < 0.05 were considered significant.

## Results and discussion

The objective of the study was to identify the pathways used by *P. freudenreichii* to cope with the different stresses encountered in the colon. A transcriptomic analysis of *Propionibacterium freudenreichii* (i) kept for 24 H in the colon of piglet (intra-colonic environment) or (ii) kept in spent YEL culture medium was performed. A dialysis bag containing *P. freundenreichii* (10^10^ CFU/mL) in stationary phase was introduced for 24 H in the colon of four piglets through a cannula implanted surgically 7 days earlier. This 24 H –period was compatible with pig colonic transit time. This was repeated on four consecutive days, a time to take into account intra-animal variability. After each 24 h incubation period in the intra-colonic environment, the content of the dialysis tubing was used for enumerations and RNA extraction. Four RNA (out of 16) were excluded from subsequent analysis because of poor quality or quantity (see Additional file 4: Table S2 for details). An 8*15 K Agilent microarray was designed especially for this experiment and hybridized with cDNA from the retro-transcription of RNA extraction. The control was RNA extracted from bacteria in stationary phase kept in the spent medium. Twelve RNA from colonic environment and twelve RNA from spent YEL medium were hybridized on microarrays. For the intra-colonic environment, eight samples out of twelve gave the same response profile (Additional file 7: Figure S3). We have chosen to analyze the transcriptome of these eight samples. A modified *t*-test was performed to list differentially expressed genes between samples from the intra-colonic environment and the control (spent medium). Most differentially expressed genes were confirmed with RT quantitative PCR (RT-qPCR) on RNA from three biological replicates from colonic environment and spent medium as control. RT-qPCR always confirmed the induction or repression of gene per microarray but standard deviation (individual variability) was greater with RT-qPCR than with microarray data.

**Figure 1:**
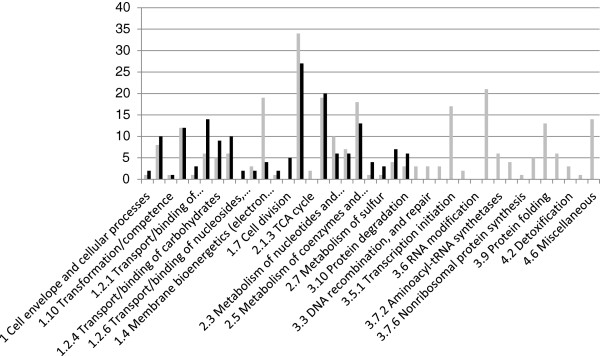
**Number of induced and repressed genes in the colonic environment, compared to spent medium, classified by metabolic function.** In grey and in black: induced and repressed genes, respectively. Differential comparisons between groups were performed gene by gene using a modified *t*-test. Genes were declared as differentially expressed (DE) with a P value ≤ 0.001 and |fold change| > 2.

Overall, 767 genes (357 genes induced (Table 1, Additional file 8: Table S4), 410 genes repressed (Table 2, Additional file 9: Table S5) were declared as differentially expressed. (Complete microarray expression data for these genes available on GEO, Genbank: GSE50089). The differentially expressed genes represented 31% of the protein-coding genes of the CIRM-BIA1^T^ genome targeted in the microarray. Category of genes most affected by intra-colonic environment was those genes implicated in carbohydrates metabolism (with 27 genes of this functional category induced and 34 genes repressed). Amino acid metabolism was the second most affected category with 19 repressed and 20 induced genes. Lastly, two categories were highly repressed: ribosomal proteins with 21 genes repressed and transcription initiation with 17 genes repressed (Figure 1).

**Table 1:** List of induced genes in intra-colonic environment compare to spent medium with RT-qPCR confirmation

**Locus tag**	**Gene**	**Descript**	**Function**	**FC microarray (induced in colon)**	**p value microarray**	**FC RT-qPCR (induced in colon)**	**pvalue RT- qPCR**	**Probe name**
PFREUD_23410		Permease of glycerol 3 P ABC transporter	1.2 Transport/binding proteins and lipoproteins	4,8	5,0E-08	34,0	1,4E-01	CUST_2220_PI426428742
PFREUD_23760		Xanthine/uracil permease	1.2 Transport/binding proteins and lipoproteins	4,5	6,0E-08	3,7	9,0E-02	CUST_2250_PI426428742
PFREUD_06780		ATP-binding protein of iron compound ABC transporter	1.2.3 Transport/binding of inorganic ions	5,4	7,7E-08	5,1	1,9E-01	CUST_662_PI426428742
PFREUD_16350		Siderophore exporter 2.A.1.38.2	1.2.3 Transport/binding of inorganic ions	4,4	1,3E-07	1,4	2,5E-01	CUST_1559_PI426428742
PFREUD_02570	*fepC*	Permease component of iron ABC transporter	1.2.3 Transport/binding of inorganic ions	3,5	1,7E-07	3,3	3,1E-01	CUST_254_PI426428742
PFREUD_02700	*glpT*	Glycerol-3-phosphate transporter	1.2.4 Transport/binding of carbohydrates	3,1	4,1E-07	1,1	2,6E-01	CUST_267_PI426428742
PFREUD_06660		IM protein of ribose/xylose/arabinose/galactoside ABC transporter	1.2.4 Transport/binding of carbohydrates	5,2	1,1E-08	1,2	1,3E-01	CUST_650_PI426428742
PFREUD_12690	*ydaO*	IM protein of branched-chain amino acids ABC transporter (HAA: undef: Branched-chain amino acids)	1.2.5 Transport/binding of amino-acids	5,7	2,0E-08	3,7	2,0E-01	CUST_1218_PI426428742
PFREUD_03350	*cytX*	Hydroxymethylpyrimidine transporter CytX	1.2.6 Transport/binding of nucleosides, nucleotides	4,0	1,7E-06	1,4	4,0E-01	CUST_332_PI426428742
PFREUD_15480	*ftsQ*	Cell division protein FtsQ	1.7 Cell division	4,1	3,0E-07	1,5	3,5E-01	CUST_1474_PI426428742
PFREUD_19110	*iolT3*	iolT3 (myo-inositol transporter iolT3)	2.1 Metabolism of carbohydrates and related molecules	3,4	7,0E-08	5,9	2,8E-02	CUST_1811_PI426428742
PFREUD_19080	*iolB*	iolB (Myo-inositol catabolism IolB protein)	2.1.1 Specific carbohydrate metabolic pathway	2,4	3,2E-07	1,9	4,3E-01	CUST_1808_PI426428742
PFREUD_22850	*gntP*	Gluconate transporter (transmembrane)	2.1.1 Specific carbohydrate metabolic pathway	3,8	3,1E-06	24,1	5,0E-03	CUST_2167_PI426428742
PFREUD_04270	*eda, hga, kdgA*	2-dehydro-3-deoxyphosphogluconate aldolase/4-hydroxy-2-oxoglutarate aldolase	2.1.1 Specific carbohydrate metabolic pathway	2,1	2,6E-05	2,8	2,0E-01	CUST_421_PI426428742
PFREUD_07170	*pccB*	Propionyl-CoA carboxylase beta chain	2.1.1 Specific carbohydrate metabolic pathway	2,5	8,0E-08	1,1	3,2E-02	CUST_701_PI426428742
PFREUD_09060	*dhaG*	Glycerol dehydratase reactivation factor DhaG	2.1.1 Specific carbohydrate metabolic pathway	3,8	5,6E-07	4,9	2,5E-02	CUST_877_PI426428742
PFREUD_09130	*pduP*	CoA-dependent propionaldehyde dehydrogenase PduP	2.1.1 Specific carbohydrate metabolic pathway	5,8	7,7E-08	4,7	4,0E-02	CUST_884_PI426428742
PFREUD_12840	*ldh2*	L-lactate dehydrogenase	2.1.2 Main glycolytic pathways	3,6	8,3E-07	1,0	1,5E-01	CUST_1233_PI426428742
PFREUD_07770	*araD*	L-ribulose-5-phosphate 4-epimerase AraD	2.1.2 Main glycolytic pathways	3,3	4,2E-08	4,7	5,9E-02	CUST_759_PI426428742
PFREUD_11340	*metH*	Methionine synthase (5-methyltetrahydrofolate:L-homocysteine S-methyltransferase)	2.2 Metabolism of amino acids and related molecules	2,1	9,2E-07	5,0	1,6E-01	CUST_1091_PI426428742
PFREUD_12630	*cysK*	Cysteine synthase (O-acetylserine sulfhydrylase)	2.2 Metabolism of amino acids and related molecules	2,1	7,9E-07	5,3	1,2E-01	CUST_1214_PI426428742
PFREUD_13940	*argH*	Argininosuccinate lyase (Arginosuccinase)	2.2 Metabolism of amino acids and related molecules	2,3	1,2E-06	2,5	1,3E-01	CUST_1323_PI426428742
PFREUD_02210	*bkdB*	Dihydrolipoyllysine-residue (2-methylpropanoyl)transferase.	2.2 Metabolism of amino acids and related molecules	2,6	2,9E-06	5,5	4,0E-02	CUST_214_PI426428742
PFREUD_03880	*ask*	Aspartokinase (Aspartate kinase)	2.2 Metabolism of amino acids and related molecules	3,3	2,5E-07	1,3	1,8E-01	CUST_383_PI426428742
PFREUD_11270	*cmk*	Cytidylate kinase (CK) (Cytidine monophosphate kinase) (CMP kinase)	2.3 Metabolism of nucleotides and nucleic acids	2,5	9,4E-07	30,0	8,0E-05	CUST_1084_PI426428742
PFREUD_00670		1-acyl-sn-glycerol-3-phosphate acyltransferase	2.4 Metabolism of lipids	4,2	1,1E-08	1,5	3,9E-01	CUST_66_PI426428742
PFREUD_07140		NUDIX hydrolase	2.6 Metabolism of phosphate	3,0	2,1E-07	4,1	2,5E-02	CUST_698_PI426428742
PFREUD_21590	*cysW*	Sulfate transport system permease protein CysW	2.7 Metabolism of sulfur	4,1	3,6E-07	14,3	2,5E-02	CUST_2047_PI426428742
PFREUD_11100		Single-strand binding protein/Primosomal replication protein	3.1 DNA replication	2,6	1,3E-05	63,5	1,6E-01	CUST_1071_PI426428742
PFREUD_01220		DNA polymerase	3.1 DNA replication	2,3	1,7E-07	3,7	9,0E-02	CUST_124_PI426428742

**Table 2:** List of repressed genes in intra-colonic environment compared to spent medium with RT-qPCR confirmation

**Locus tag**	**Gene**	**Descript**	**Function**	**FC microarray (induced in colon)**	**pvalue microarray**	**FC RT-qPCR (induced in colon)**	**pvalue RT- qPCR**
PFREUD_14940	*sufS*	Cysteine desulphurases, SufS	1.2 Transport/binding proteins and lipoproteins	7,4	2,5E-07	1,4	6,9E-02
PFREUD_19650	*feoB*	Ferrous iron uptake protein B 9.A.8.1.x	1.2.3 Transport/binding of inorganic ions	6,7	1,0E-07	1,3	3,5E-02
PFREUD_20460	*cycA1*	D-serine/D-alanine/glycine transporter 2.A.3.1.7	1.2.5 Transport/binding of amino-acids	16,5	3,9E-05	2,0	2,4E-02
PFREUD_05220	*nuoG*	NADH-quinone oxidoreductase chain G	1.4 Membrane bioenergetics (electron transport chain and ATP synthase)	9,9	4,3E-08	5,3	6,0E-03
PFREUD_10590	*mcoE*	Methylmalonyl-CoA epimerase	2.1.1 Specific carbohydrate metabolic pathway	9,3	3,0E-07	21,7	6,8E-02
PFREUD_01040	*gntK*	Gluconate kinase (Gluconokinase)	2.1.1 Specific carbohydrate metabolic pathway	2,9	2,9E-05	39,5	4,3E-02
PFREUD_18870		Methylmalonyl-CoA carboxytransferase 5S subunit.	2.1.1 Specific carbohydrate metabolic pathway	10,3	1,1E-07	10,0	1,3E-02
PFREUD_17320	*eno1*	Enolase 1	2.1.2 Main glycolytic pathways	8,4	3,6E-07	26,3	2,0E-03
PFREUD_23890	*fba2*	Fructose-bisphosphate aldolase class I	2.1.2 Main glycolytic pathways	21,3	1,1E-08	68,6	2,0E-03
PFREUD_05650	*tuf*	Elongation factor Tu	3.7.4 Translation elongation	4,8	4,8E-05	2,0	4,9E-01
PFREUD_17840	*dnaK1*	Chaperone protein dnaK 1	3.9 Protein folding	15,5	1,5E-07	23,3	2,5E-03
PFREUD_19250	*clpB 1*	Chaperone clpB1	3.9 Protein folding	3,6	4,1E-04	10,9	6,6E-02
PFREUD_22780	*hsp20 1*	Heat shock protein 20 1 (20 kDa chaperone 1)	3.9 Protein folding	36,6	2,5E-08	20,2	1,4E-02

1-Down regulation of glycolysis, Wood-Werkman cycle and oxidative phosphorylation

In YEL medium, the glycolysis, the pentose phosphate pathway and the Wood Werkman cycle (fermentation) are three ways for *P. freudenreichii* to produce NADH and NADPH reducing equivalents, ATP and precursor metabolites needed for the biosynthesis of essential compounds (amino acids, purine, pyrimidine, glycerol 3 phosphate, fatty acids, N-acetyl glucosamine, vitamins). In the intra-colonic environment, glucose is lacking and the bacteria are in anaerobic conditions. Microarray analysis revealed a down regulation of genes involved in glycolysis, the Wood-Werkman cycle and oxidative phosphorylation. All the genes encoding proteins involved in glycolysis were repressed (Additional file 10: Figure S4). Some of them were particularly down-regulated: *pgm* with a fold change of -5.9, *fba2* with a fold change of -21.5 (confirmed by RT-qPCR with a fold change of -145.7), *gap* with a fold change of -11.5 and *eno1* with a fold change of -8.4 (confirmed by RT-qPCR with a fold change of -50.7). The Wood-Werkman cycle (Additional file 10: Figure S4) is specific to some propionic acid producing bacteria. It holds a central place in propionic fermentation, the main central carbon metabolic pathway in dairy propionibacteria. By this pathway, pyruvate is converted into propionate. Pyruvate is first converted to succinate by successive steps of the tricarboxylic cycle (TCA). All corresponding genes were repressed in the intra-colonic environment. Succinate is then converted into succinyl CoA, methylmalonyl CoA, propanoyl CoA and propionate by specific enzymes, whose corresponding transcripts were down-regulated. *mutA* and *mutB* were repressed with fold changes of -5.8 and -4.8, respectively, *mcoE* was repressed with a fold change of -9.3 (confirmed with a fold change of -21.7, p-value=0.06, by RT-qPCR). Transcripts corresponding to the 12S and 5S subunits (no probe was designed for the 1.3 S subunit) of the well-studied methylmalonyl-CoA carboxytransferase were repressed with fold changes of -7 and -10.3, respectively. The latter repression was confirmed with a fold change of -20.1 by RT-qPCR. The down-regulation of Wood-Werkman cycle is probably involved in maintaining the redox balance which is drastically affected by intra-colonic environment [41] and is probably a consequence of the low availability of pyruvate in intracellular content. Although glycolysis and the Wood Werkman cycle were down-regulated, all the genes of the pentose phosphate pathway (except *gnd2* gene) were stably expressed. This pathway produces a major source of reducing equivalent: NADPH needed for biosynthesis reactions and NADH necessary for oxidative phosphorylation.

Several transcripts involved in aerobic respiration were down-regulated in the intra-colonic environment: *nuoG* encoding the G chain of NADH-quinone oxydoreductase (responsible for the release of electrons and H+ contained in NADH molecule) with a fold change of -9.9 (confirmed with a fold change of -14.2 by RT-qPCR); electron transfer flavoprotein-quinone oxydoreductase *fixC* and *fixB* with fold changes of -4.3 and -2.2, respectively; *cydB* and *cydA* encoding cytochrome d ubiquinol oxidase subunit I and II with fold changes of -3.5 and -6.6, respectively. These latter results were impossible to confirm using RT-qPCR because of the difficulty of targeting one specific transcript among multi-genic family transcripts. On the otherhand, some transcripts specific to anaerobic respiration were induced. Genes *dmsA* and *dmsB* encoding anaerobic dimethyl sulfoxide reductase, chain A and B were induced with fold changes of +3.2 and +2.5, respectively. The latter protein is responsible for the final transfer of electrons on various sulfoxide and N-oxide compounds. Another electron sink could be ferrous ion or sulfur. Accordingly, the gene encoding the sulfur transporter *cysW* was induced with a fold change of 4.1 (confirmed by RT-qPCR with a fold change of 9.2). Anaerobiosis-inducible dimethyl sulfoxide reductases play a key role in bacterial adaptation to anaerobic conditions in bacteria and serve as terminal reductases using DMSO as a terminal electron acceptor. They are involved in the colonization of intestine by Campylobacter jejuni [42] and *dmsA* mutation results in reduced in vivo adaptation in Actinobacillus pleuropneumoniae [43]. Such induction in *P. freudenreichii* reveals efficient adaptation to the anaerobic conditions of the colon content. Similarly, an IVET approach identified a methionine sulfoxide reductase involved in adaptation and persistence in the murine gut [27].

The observed changes suggest profound metabolic reprogramming in response to the intra-colonic environment. Such reprogramming has been described in *Bifidobacterium longum*, where exposure to bile salts in vitro [44] or to the colon in vivo [45] induces expression of the fructose-6-phosphate phosphoketolase (F6PPK, or bifid shunt pathway) at the expense of other catabolic pathways. Similarly, in the present study, specific catabolic.

2-Induction of specific carbohydrate catabolism and alternative pathways to produce NADH, NADPH, ATP and precursors.

If the intra-colonic environment is particularly glucose-depleted because of massive absorption upstream in the upper part of the gut during digestion, other carbohydrates like lactate, gluconate and propanediol are produced *in situ* by autochthonous bacteria of the colon. *P. freudenreichii* CIRM BIA1 has the ability to degrade these sugars [32]. Microarray analysis revealed the ability of *P. freudenreichii* to induce *in vivo* these peculiar catabolic pathways to generate reducing equivalents, ATP and metabolic precursors.

a. Lactate

Expression of the *ldh*1 and *ldh*2 genes, both encoding L-lactate dehydrogenase was enhanced with a fold change of +2.2 and +3.6, respectively (not confirmed by RT-qPCR), while D-lactate dehydrogenase *dld* was repressed with a fold change of −2.5. Although not a major fermentation product in the gut, lactate is an important electron sink in the colonic environment [46]. Both D- and L-stereoisomers exist in the colon, in concentrations on the millimolar scale. L-lactate is more abundant and is produced by many enteric bacteria including Bifidobacteria, Streptococci, Enterococci and Clostridia. D-lactate is a minor fermentation product in the colon. Differential modulation of L-lactate at the expense of D-lactate dehydrogenases could indicate metabolic adaptation to the colon setting, considering that lactate, including both stereoisomers, is the preferred carbon source for *P. freudenreichii* in fermented dairy products.

b. Gluconate

**Figure 2:**
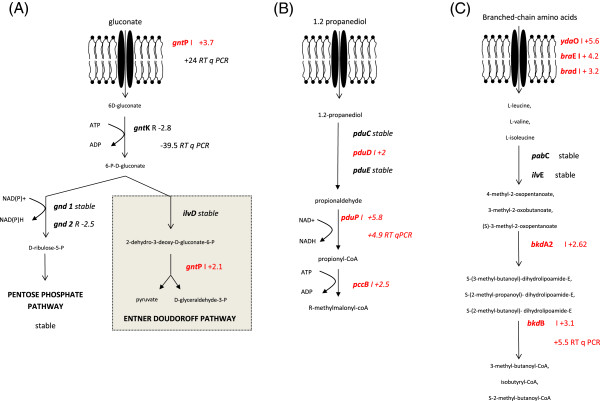
**Induction of gluconate, propanediol and branched-chain amino acid degradation pathway in the colonic environment compared to spent medium. (A)** Induction of gluconate degradation pathway in the colonic environment compared to spent medium. I and R indicate induced and repressed genes in the colonic environment, compared to spent medium, followed by the fold change based on a mean of four repetitions of microarray data. Differential comparisons between groups were performed gene by gene using modified *t*-test. Genes were declared as differentially expressed with a p-value ≤ 0.001 and |fold change| > 2. Three repetitions of RT-qPCR were performed and genes were declared as DE with a Student test P value < 0.05. **(B)** Induction of propanediol degradation in the colonic environment, compared to spent medium. Differential comparisons between groups were performed gene by gene using modified *t*-test. Genes were declared as differentially expressed with a P value ≤ 0.001 and |fold change| > 2. R indicates a repression. I indicates an induction. Numbers indicate the fold change based of a mean of four repetitions for microarray data. Three repetitions of RT-qPCR were performed and genes were declared as DE with a Student test p-value < 0.05. **(C)** Pathway of amino acid catabolism induced in the colonic environment, compared to spent medium. Differential comparisons between groups were performed gene by gene using a modified *t*-test. Genes were declared as differentially expressed with a P value ≤ 0.001 and |fold change| > 2. I indicates an induction. Numbers indicates the fold change based on a mean of four repetitions for microarray data. Three repetitions for RT-qPCR were performed and genes were declared as differentially expressed with Student test p-value < 0.05.

Gluconic acid is another intestinal substrate that can be degraded by *P. freudenreichii*. It is an organic compound present in fruits and other vegetable products, but also in the intestinal mucus, which represents a major source of carbohydrates for enteric bacteria [47]. In *P. freudenreichii*, it is also a possible carbon and energy source and the gluconate degradation pathway was accordingly induced in the intra-colonic environment (Figure 2). The degradation of gluconate by entire cells *of P. freudenreichii* has, to our knowledge, not been previously mentioned. The metabolic pathway was reconstructed following an *E. coli* model. The gene *gntP* coding for the gluconate transporter was induced 3.7 fold in the intra-colonic environment compared to the YEL medium. The induction was confirmed with a fold change of +24 using RT-qPCR. The second step of the gluconate catabolic pathway is the phosphorylation of gluconate into 6P gluconate by glucokinase. Surprisingly, the expression of *gntK* was down regulated with a fold change of −2.8 (confirmed by RT-qPCR with a fold change of - 39.5). We speculate that this phosphorylation step could be performed by another non-specific carbohydrate kinase such as PFREUD_03250 (annotated as *rbsK*) induced with a fold change of +3.6 in the intra-colonic environment. 6P gluconate is then degraded by the pentose phosphate pathway the genes of which were stably expressed (*gnd*, *rpiB*, *tal* genes, see above) or by the Entner Doudoroff pathway. Gene *eda* of the latter pathway was induced with a fold change of 2.1 (not confirmed statistically by RT-qPCR). Pentose phosphate and Entner Doudoroff pathways produce NADPH which can be used for further biosynthesis reactions.

c. Propanediol

Propanediol is another substrate available for bacteria in the anaerobic environment of the colon because it is produced by the degradation of rhamnose and fucose by the commensal microbiota [48,49]or by the degradation of glycerol by *Clostridium* and *Enterobacteriacea*[50]. Propanediol degradation produces NADH which can be used as a source of electrons for oxidative phosphorylation (Figure 2). A locus containing 15 genes involved in propanediol degradation was found in the genome of *P. freudenreichii* CIRM-BIA1^T^[32]. Most genes of this locus appeared to be induced in the intra-colonic environment: *pduD* which encodes a subunit of B12 dependent dioldehydratase, the first enzyme of propanediol degradation was induced 2-fold; *pduP* responsible for the degradation of propionaldehyde to propionyl-CoA was induced with a fold change of +5.8 (confirmed by RT-qPCR with a fold change of 4.7). Two slightly different pathways were proposed for propanediol degradation in *Salmonella typhimurium*[49] and in *Listeria innocua*[48]. All the coding sequences of the propanediol locus in *Propionibacterium freudenreichii* are highly similar to those of *Clostridium*, *Salmonella* and *Listeria* species. However, two genes (*pduL* and *pduW*) present in *Salmonella typhimurium* and *Listeria innocua* are missing in the genome of *Propionibacterium freudenreichii* CIRM-BIA1. Their products are responsible for the conversion of propionyl-CoA into propionate. We propose that the next degradation step could be operated by the enzyme encoded by *pccB* responsible for the conversion of propionyl-CoA to R-methylmalonyl-CoA which was induced with a fold change of + 2.5. R-methylmalonyl-CoA can be further degraded during the Wood-Werkman cycle.

The *pdu* operon is a key feature in the probiotic *Lactobacillus reuteri*, involved in growth on propanediol, involved in reuterin biosynthesis, in colonization of the human digestive tract [51] and in protection towards *Salmonella typhimurium* colonization [52]. To our knowledge, *L. reuteri* and *P. freudenreichii* are the only probiotic species with a *pdu* operon allowing utilization of propanediol. This ability confers an advantage to the strain. Since *pdu* mutation confers a virulence defect to *Salmonella enterica*[53] in mice, we can suppose that the catabolism of propanediol is essential for the persistence of this pathogen in their host. As a consequence, *P. freudenreichii* can also be considered to compete with *Salmonella enterica* for utilization of the propanediol present in the intestine.

d. Metabolism of amino acid

Intra-colonic conditions affected amino-acids transport and catabolism pathways. The *dcuA* gene, encoding a C4-dicarboxylate uptakeproteins responsible for the transport of aspartate, was repressed with a fold change of −5.2 (Additional file 9: Table S5). The genes *cycA1* and *cycA2*, the products of which are responsible for the transport of glycine and alanine, were repressed with a fold change of −16.5 and −6.6, respectively (*cycA1* repression was confirmed by RT-qPCR with a fold change of −2). The gene encoding L- aspartate oxidase (*nadB2*) was repressed with a fold change of −9.3, consistent with the absence of oxygen, which is essential to its catabolic reaction. Repression of *dadA2* and *ald*, with a fold change of −3.4 and −61, indicates a down-regulation of glycine and alanine degradation, respectively.

On the other hand, other transcripts involved in aspartate degradation like *aspB*, *tyr*B and *got* were slightly induced with fold changes of +2.3, 1.7 and 2.1, respectively.

Similarly, (Figure 2) three genes encoding the inner membrane protein component of branched-chain amino acids ABC transporter were induced in the intra-colonic environment: *ydaO*, *braE* and *brad* with fold changes of +5.6, 4.2 and 3.2, respectively. These branched-chain amino acids can be introduced into proteins or transformed into enoyl-CoA, incorporated into branched chain fatty acids. All genes corresponding to the degradation pathway that produces enoyl CoA were stably expressed or induced in the intra-colonic environment. *bkdA2* and *bkdB* were induced with fold changes of 2.6 and 2.8, respectively (*bkdB* was confirmed with a fold change of 5.5 by RT-qPCR). Branched chain fatty acids can be introduced in the bacterial membrane, conferring fluidity and resistance to oxidation, or released into the lumen. A protective role of BCFAs in intestinal barrier function of the host has recently been described [54].

e. Purine and pyrimidine

*P. freudenreichii* also seems to use ribose and nucleotides as sources of carbon and nitrogen. The degradation pathway of uracil, an RNA-specific base, was induced. The uracil transporter (Xanthine/uracil permease) encoded by PFREUD_23760 was induced +4.5 (confirmed with a fold change of +3.7 by RT-qPCR). The gene *cmk* encoding a cytidylate kinase was induced +2.5 (confirmed with a fold change of +30 by RT-qPCR). Purine and pyrimidine are available in the intestine as a result of the degradation of nucleic acids obtained from diet. Bases are required by *E. coli* to colonize of the intestine [55]. The ability to use such bases to adapt to the colonic conditions seems to be shared by *P. freudenreichii*.

3-Recovery of precursor metabolites in the medium to fulfill anabolic needs

Transcript corresponding to the transport of inositol *iolT3* was induced +3.4 (confirmed with a fold change of +5.9 by RT-qPCR) whereas the remaining degradation pathways were slightly induced (*iolD* +1.5 and *iolB* +2.4) or stably expressed. Inositol is a carbohydrate found in many vegetable food components, including fruits but also cereals, nuts and beans, which were present in the pig diet in this study. Its bioavailability is enhanced by degradation of phytates within the gut lumen by members of the gut microbiota [56]. Inositol thus constitutes a usable substrate for *P. freudenreichii* in the gut environment.

Similarly, the glycerol and glycerol 3P transporter PFREUD_23410 was induced +4.7 (confirmed with a fold change of +34 by RT-qPCR), whereas the degradation pathway of glycerol was not induced. Glycerol is another substrate available in the gut as a result of the hydrolysis of dietary triglycerides by digestive enzymes. *P. freudenreichii* can ferment glycerol, a property used in the LGA selective medium for the isolation of dairy propionibacteria [57].

Both inositol and glycerol are incorporated into *P. freudenreichii* membranes in the form of phosphatidyl inositol. However, this anabolic pathway is not induced, as indicated by the repression of the phosphatidyl inositol transferase encoded by *pgs*1 with a fold change of −1.5 (p-value < 0.001, data not shown). Glycerol and inositol are thus most likely used partly as carbon and energy sources in the intestine and partly incorporated as cell building blocks. We suspected that glycerol is also needed during propanediol degradation, interacting with DhaG a glycerol dehydratase reactivation factor encoded by a gene *dhaG* induced with a fold change of +3.8 (confirmed by RT-qPCR with a change of +4.9) and present in the middle of propanediol operon.

**Figure 3:**
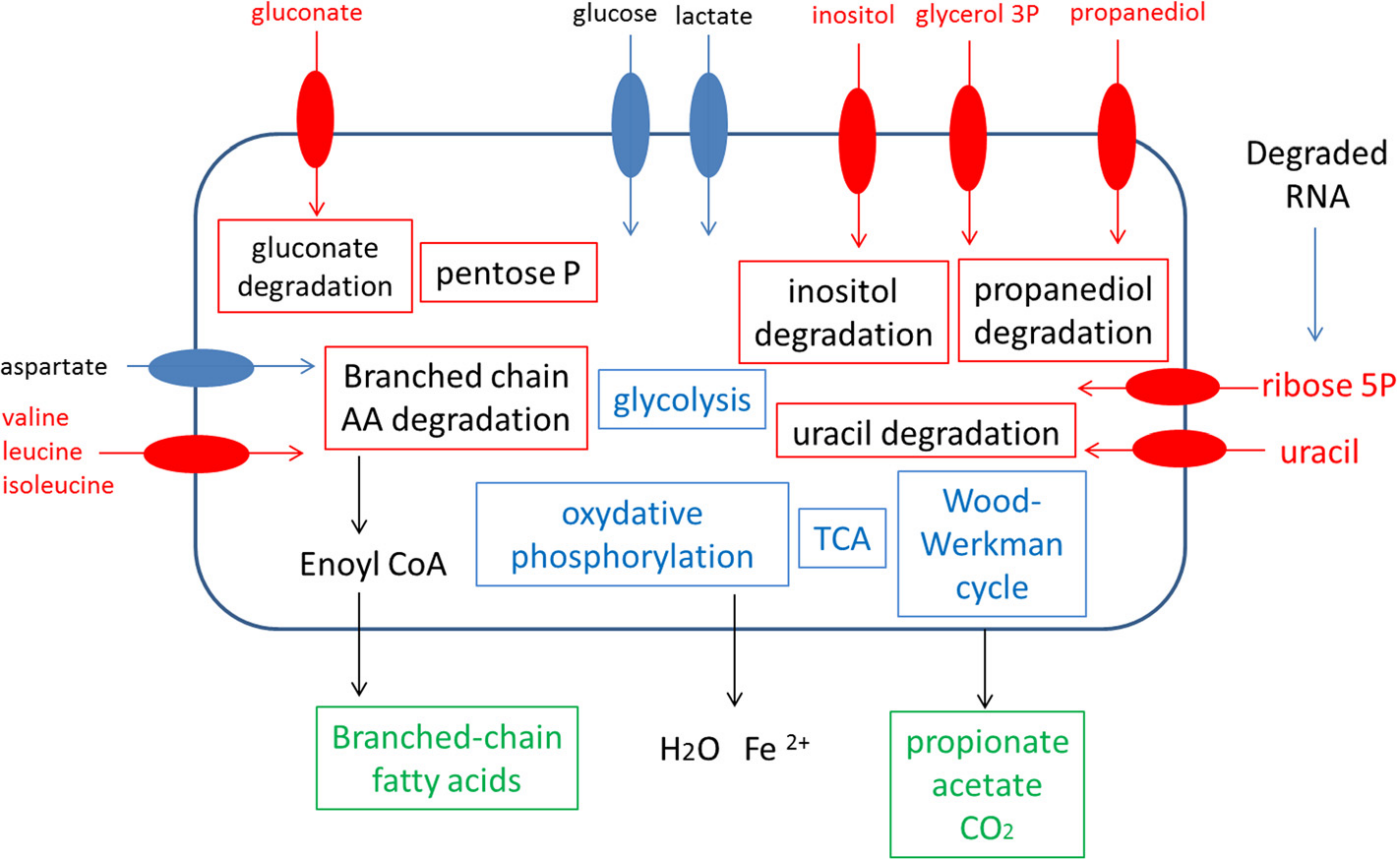
**Overview of *Propionibacterium freudenreichii *metabolism of in the colonic environment compared to spent medium.** Differential comparisons between groups were performed gene by gene using a modified *t*-test. Genes were declared as differentially expressed with P value ≤ 0.001 and |fold change| > 2. In blue, genes repressed in the colonic environment: glycolysis, tricarboxylic acid cycle (TCA), Wood-Werkman cycle, oxidative phosphorylation. In red, gene induced or stably expressed in the colonic environment compared to spent medium: pentose phosphate, gluconate, inositol, propanediol, uracil and branched-chain amino-acid degradation.

Figure 3 synthetizes all induced and repressed catabolic pathways of cells in colonic environment compared to *P. freudenreichii* cells maintained in the spent medium.

4-Down regulation of protein synthesis but induction of genes involved in cell division

Protein production is probably reduced. It is consistent with the repression of several ribosomal proteins and the *tuf* gene, which encodes the translation elongation factor (Additional file 9: Table S5) with a fold of – 4.8. Counts before introduction into the colon and after 24H in the colon showed a good survival rate (66%) with high repeatability (1.2 E^10^ +/− 1.6 E^9^ before versus 8 E^9^ +/− 1.6 E^9^ CFU after 24H, significant *t*-test p-value < 0.001). Nevertheless, microarray showed induction of genes expressed specifically during cell division: *ftsE*, *ftsQ*, *ftsW1*, *ftsW2* with a fold change of +3.3, +4, +2.3, +2, respectively and in DNA replication: *dnaA* encoding a chromosomal replication protein, PFREUD_21610 encoding a primosomal replication protein, *dna*E1 encoding the alpha subunit of polymerase III, PFREUD_01220 encoding a DNA polymerase and *polA* encoding the DNA polymerase I were induced with a fold change of +2, +2.6, +2.2, +2.3 and +2.3, respectively (Additional file 8: Table S4). This suggests that part of the propionibacteria population recovered the ability to divide after 24H in the colon.

5-Down regulation of general stress proteins

Considering that the colonic environment is thought to be stressful, molecular chaperones were surprisingly repressed in *P. freudenreichii* in this condition, compared to spent YEL medium. For example, expression of GroEL and GroES is generally induced in bacteria by different stresses that lead to protein misfolding. In *P. freudenreichii*, pulse labelling and proteomic analysis revealed induction of this complex by acid stress [21], yet not by thermal and bile salts stress [23,24]. It is repressed here under intra-colonic conditions, suggesting that no major protein misfolding is sensed by *P. freudenreichii*. Moreover, both *dnaJ1* and *dnaJ2* molecular chaperones, as well as *hsp*20 which is involved in disaggregation of stress-denatured proteins, were repressed with fold changes of −2.8, -3.9 and −36, respectively (confirmed with a fold change of −20 by RT-qPCR). The same was observed for the stress-induced ATP-dependent proteinase *clpB* (−3.6 confirmed by RT-qPCR with a fold-change of −10.9, while clpC was non-significantly repressed, -2.17 fold with p-value = 0.055) involved in stress-denatured proteins degradation and turnover, for the chaperones *dnaK1* (−15.5 confirmed at −23.3 by RT-qPCR) and *dnaK2* (−7.7) and for the co-chaperone *grpE1* (−5.5) and *grpE2* (−3.5). By contrast, an R-IVET approach showed *clpC* to be involved in digestive adaptation in *L. plantarum* WCFS1 [27]. Together, these data suggest that no major damage at the level of protein folding and function is sensed by *P. freudenreichii* in colonic conditions compared to the control culture left in spent medium. The repression of chaperones agrees with the abundance of carbon and nitrogen exploited by *P. freudenreichii* in the colonic environment (see above).

6-Limit of the method

We designed an innovative approach to tackle the question of adaptation of a probiotic to the colonic environment. However, although innovative, this approach has several drawbacks that must be acknowledged. The first one is that the transcriptomic profile we observed for *Propionibacterium freudenreichii* within the gut is probably related to the specific microbiota of our pigs. In particular, it must be acknowledged that following surgery, pigs received intra-muscular ampicillin for 3 consecutive days. This probably modified gut microbiota as demonstrated by Janczyk et al. 2007 [58] who observed long-lasting effect of amoxicillin on some bacteria abundance in piglets (decrease of *Roseburia faecalis*-related population and increase of an enterobacterial population with 100% identity to *Shigella* spp., *Escherichia coli* and *Salmonella enterica* serovar *Typhi*). However other studies demonstrated microbiota resilience within few days in other species [59]. Similarly, the influence of the pig diet on gut microbiota and substrate availability must be acknowledged. The other drawback is the influence of pig diet since we can imagine that a higher supply of propanediol and gluconate would have enhanced *P. freudenreichii* probiotic potential. Despite these limitations in the approach, we believe our data will greatly benefit the scientific community working on probiotic bacteria and *P. freudenreichii* specifically. Further studies using the same innovative approach but comparing Propionibacteria transcriptomic profile in pigs harboring different microbiota composition (whether due to antibiotic treatment, diet composition or any other factor influencing microbiota composition) would constitute another step into the understanding of probiotic action upon gut and host physiology but are beyond the scope of this study.

## Conclusions

The aim of this study was to obtain a molecular insight into the adaptation of *P. freudenreichii* to intra-colonic conditions. Transcriptomic analysis revealed metabolic reorientation in accordance with intestinal conditions (Figure 3). Glycolysis, the Wood-Werkman cycle and oxidative phosphorylation were probably down-regulated (in blue) due to the lack of glucose and anoxic conditions encountered in the intra-colonic environment. However, *P. freudenreichii* was able to induce pathways (in red) involved in the metabolism of substrates available in the colon (propanediol, gluconate, inositol, amino-acids and nucleic bases) and in the production of energy (ATP), reducing equivalent (NADH and NADPH) and metabolic precursors. Furthermore, induction of genes indicating growth (cell division proteins), as well as DNA polymerase involved in replication, confirmed the adaptation 24H after the introduction of *P. freudenreichii* into the pig colon. Survival and metabolic activity within the gut, which constitute a prerequisite to *P. freudenreichii* probiotic potential expression, seem to be achieved by utilization of specific substrates such as propanediol and gluconate. This unique *in vivo* experimental approach is the first demonstration of *P. freudenreichii* physiological adaptation to the colon of an omnivorous mammal.

## Competing interests

The authors declare that they have no competing interests.

## Authors’ contributions

TS developed *in vivo* used dialysis devices and carried out RNA extractions. SP carried out RNA sample labeling and microarray hybridizations. GG participated in the design of the study and performed the statistical analysis. VL created databases and implemented bioinformatics tools. JM and AL participated to microarray design, hybridizations and scanned microarrays. HF designed microarrays and reconstructed metabolic pathways. GB conceived animal experimentation and participated to the writing. HF and GJ conceived the study, and participated in its design, carried out the coordination and wrote the manuscript. All authors read and approved the final manuscript.

## Authors’ information

Gwenaël Jan and Hélène Falentin share the same seniority.

## Supplementary Material

Additional file 1: Figure S1 Dialysis device allowing development of the bacterial implant. Click here for file

Additional file 2: Table S1 Diet composition. Click here for file

Additional file 3: Figure S2 Flowchart of experiment. Click here for file

Additional file 4: Table S2 Flowchart of RNA samples. Click here for file

Additional file 5: Table S3 Primers used for reverse transcription quantitative PCR (RT-qPCR). Click here for file

Additional file 6: Figure S5 Repression of glycolysis. Click here for file

Additional file 7: Figure S3 Heatmap of statistical analysis of microarray. Click here for file

Additional file 8: Table S4 List of gene induced in colonic environment compared to spent medium with fold change >2 and p value <0,001. Click here for file

Additional file 9: Table S5 List of gene repressed in colonic environment compared to spent medium with fold change >2 and p value <0,001. Click here for file

Additional file 10: Figure S4 The Wood Werkman cycle responsible for release of propionic acid was repressed in colonic environment. Click here for file
