# Supplementary material for: A unique in vivo experimental approach reveals metabolic adaptation of the probiotic Propionibacterium freudenreichii to the colon environment
Source: BMC Genomics. 2013 Dec 23;14:911. doi: 10.1186/1471-2164-14-911 (PMC3880035; doi:10.1186/1471-2164-14-911)
Supplement: Additional file 1: Figure S1 — Dialysis device allowing development of the bacterial implant. [file 1471-2164-14-911-S1.pptx]

## Slide 1
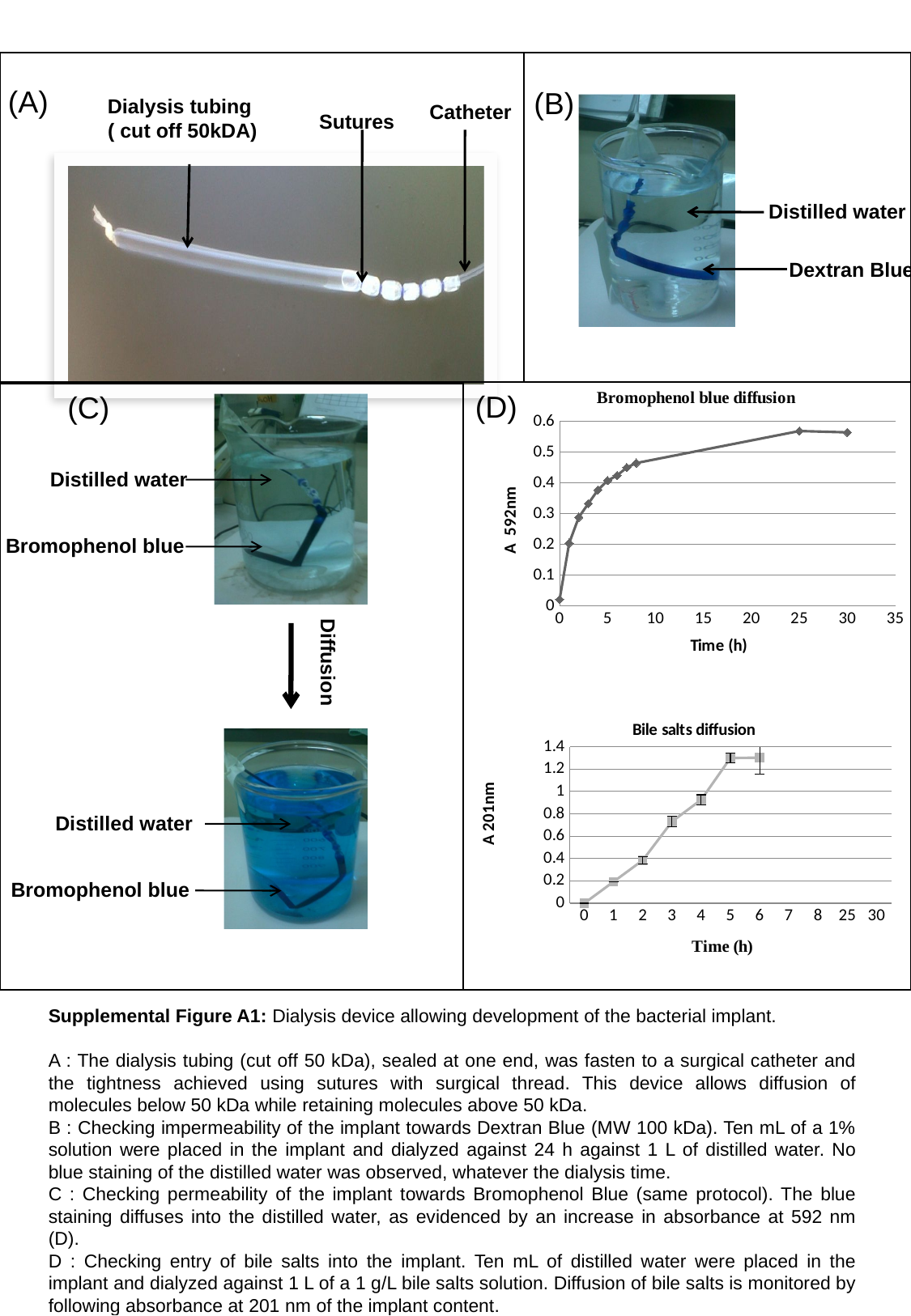

(A)
(B)
Dialysis tubing
( cut off 50kDA)
Catheter
Sutures
Distilled water
Dextran Blue
### Chart: Bromophenol blue diffusion
| Category | Do |
|---|---|(D)
(C)
Distilled water
Bromophenol blue
Diffusion
Distilled water
[unsupported chart]
Bromophenol blue
Supplemental Figure A1: Dialysis device allowing development of the bacterial implant.
A : The dialysis tubing (cut off 50 kDa), sealed at one end, was fasten to a surgical catheter and the tightness achieved using sutures with surgical thread. This device allows diffusion of molecules below 50 kDa while retaining molecules above 50 kDa.
B : Checking impermeability of the implant towards Dextran Blue (MW 100 kDa). Ten mL of a 1% solution were placed in the implant and dialyzed against 24 h against 1 L of distilled water. No blue staining of the distilled water was observed, whatever the dialysis time.
C : Checking permeability of the implant towards Bromophenol Blue (same protocol). The blue staining diffuses into the distilled water, as evidenced by an increase in absorbance at 592 nm (D).
D : Checking entry of bile salts into the implant. Ten mL of distilled water were placed in the implant and dialyzed against 1 L of a 1 g/L bile salts solution. Diffusion of bile salts is monitored by following absorbance at 201 nm of the implant content.
