# Supplementary material for: A unique in vivo experimental approach reveals metabolic adaptation of the probiotic Propionibacterium freudenreichii to the colon environment
Source: BMC Genomics. 2013 Dec 23;14:911. doi: 10.1186/1471-2164-14-911 (PMC3880035; doi:10.1186/1471-2164-14-911)
Supplement: Additional file 2: Table S1 — Diet composition. [file 1471-2164-14-911-S2.docx]

Supplemental Table : Diet composition

| Ingredients | % |
| --- | --- |
| Wheat | 23.5 |
| Corn | 25.5 |
| Barley | 24.6 |
| Soybean meal | 23.0 |
| Vegetable oil | 0.5 |
| Calcium carbonate | 1.0 |
| Monocalcium phosphate | 1.0 |
| Sodium chloride | 0.4 |
| Trace element and vitamin mix | 0.5 |
