# Supplementary figures and images for: A unique in vivo experimental approach reveals metabolic adaptation of the probiotic Propionibacterium freudenreichii to the colon environment
Source: BMC Genomics. 2013 Dec 23;14:911. doi: 10.1186/1471-2164-14-911 (PMC3880035; doi:10.1186/1471-2164-14-911)

Figure A2 : flowchart of experiment


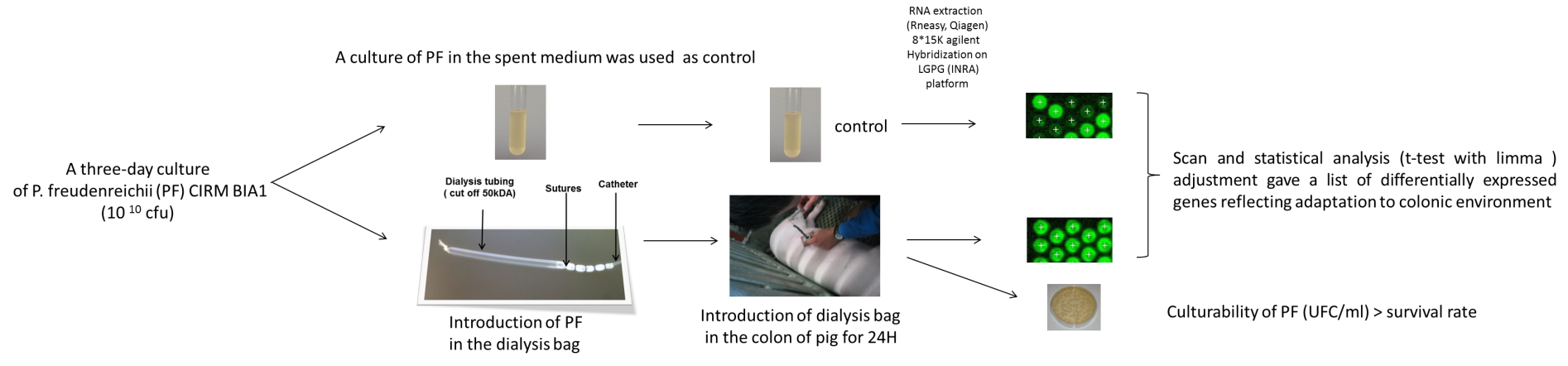

Supplement: Additional file 3: Figure S2 — Flowchart of experiment. [file 1471-2164-14-911-S3.docx]
