# Supplementary material for: A unique in vivo experimental approach reveals metabolic adaptation of the probiotic Propionibacterium freudenreichii to the colon environment
Source: BMC Genomics. 2013 Dec 23;14:911. doi: 10.1186/1471-2164-14-911 (PMC3880035; doi:10.1186/1471-2164-14-911)
Supplement: Additional file 6: Figure S5 — Repression of glycolysis. [file 1471-2164-14-911-S6.docx]

Supplemental Figure S5:

Repression of glycolysis. R indicates a repression. I indicates an induction. Numbers indicate sthe fold change based on a mean of four repetitions for microarray data and three repetitions for RT q PCR. Student test, pvalue <0.05.

***fba1*** *R -1.9*

***fba2*** *R -21.3 / R -145.7 RT q PCR*

Phosphoenolpyruvate

Glycerate 3 P

***gpm1*** *R -1.2*

***gpm2*** *R -3.7*

Glycerate 2 P

***eno1*** *R -8.4 / R -50.7 RT q PCR*

***eno2*** *R -2.5*

***pgk*** *R -2.2*

***gap*** *R -11.5*

glycerate 1,3 bi P

fructose 6 P

glyceraldehyde 3 P

***pfkA*** *I -1.7 (ATP)*

***pfp*** *R -2.2 (PPi)*

***pgi*** *R -1.5*

***pgm1*** *R -5.9*

fructose 1,6 bi P

glucose 6 P

glucose 1 P
