# Supplementary material for: A unique in vivo experimental approach reveals metabolic adaptation of the probiotic Propionibacterium freudenreichii to the colon environment
Source: BMC Genomics. 2013 Dec 23;14:911. doi: 10.1186/1471-2164-14-911 (PMC3880035; doi:10.1186/1471-2164-14-911)
Supplement: Additional file 7: Figure S3 — Heatmap of statistical analysis of microarray. [file 1471-2164-14-911-S7.docx]

Supplemental Figure A3: Heatmap of statistical analysis of microarray

Samples TS13, TS14, TS17 and TS18 were excluded from micro-array and Rt q PCR analysis because poorly correlated to other samples issued from the same conditions.


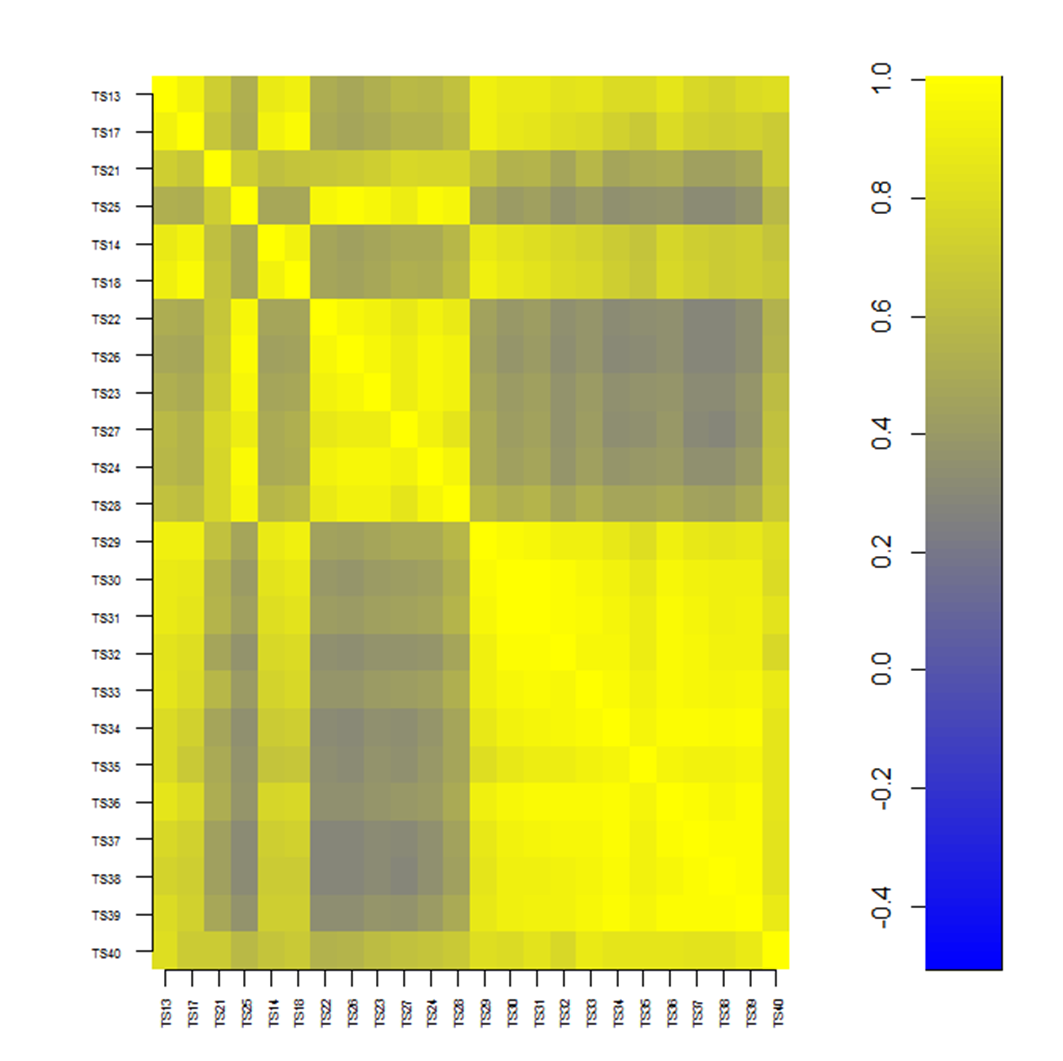


**YEL medium**

**Colonic environment**
