# Supplementary material for: A unique in vivo experimental approach reveals metabolic adaptation of the probiotic Propionibacterium freudenreichii to the colon environment
Source: BMC Genomics. 2013 Dec 23;14:911. doi: 10.1186/1471-2164-14-911 (PMC3880035; doi:10.1186/1471-2164-14-911)
Supplement: Additional file 10: Figure S4 — The Wood Werkman cycle responsible for release of propionic acid was repressed in colonic environment. [file 1471-2164-14-911-S10.docx]

Supplemental Figure A4: The Wood Werkman cycle responsible for release of propionic acid was repressed in colonic environment.

***bccp (1.3S)***

***mmcoA/mmdA (12S)*** R – 7

***PFREUD_18870 (5S)***

R – 10.2 / -20.1 RT q PCR

***sdh*** R -2/-3.5 according subunit

***mutA*** R – 5.8

***mutB*** R – 4.8

***cat*** R – 3.4

pyruvate

***mdh*** R -4.1

oxaloacetate

***mcoE*** R -9.3 / -21.7 RT q PCR

propionate

***mdh*** R -2.5

propanoyl-CoA

S-methylmalonyl-CoA

R-methylmalonyl-CoA

succinyl-CoA

succinate

fumarate

malate
